# Psychobiotic Potential of Gamma-Aminobutyric Acid–Producing Marine *Enterococcus faecium* SH9 from Marine Shrimp

**DOI:** 10.1007/s12602-022-09963-z

**Published:** 2022-06-25

**Authors:** Shimaa El Sakkaa, Eman H. Zaghloul, Khaled M. Ghanem

**Affiliations:** 1 Botany and Microbiology Department, Faculty of Science, Alexandria University, Alexandria, Egypt; 2 National Institute of Oceanography and Fisheries, NIOF, Cairo, Egypt

**Keywords:** Psychobiotic, GABA, *Enterococcus*, GAD gene, TLC, HPLC, Marine, Shrimp

## Abstract

Psychobiotics are a novel class of probiotics with potential to confer mental wellness via production of neuroactive compounds such as gamma-aminobutyric acid (GABA). The demand for new biological sources of GABA has increased steadily. Therefore, the current study reports the isolation of 17 presumptive lactic acid bacteria (LAB) from marine samples and their screening for GABA synthesis from monosodium glutamate (MSG) using thin-layer chromatography (TLC). The isolate SH9 was selected as a high GABA producing strain. The GABA content of SH9 cell free supernatant (CFS) was quantitatively determined by high performance liquid chromatography (HPLC) to be 0.97 g/L. SH9 was identified biochemically and molecularly as *Enterococcus faecium* (identity 99%). Moreover, SH9 demonstrated promising probiotic potentials; it gave no signs of hemolysis and could survive at low pH values and high bile salt concentrations. It also exhibited antimicrobial activity against highly pathogenic strains and the ability to grow at 6.5% NaCl. In addition, SH9 CFS showed anti-inflammatory and antioxidant properties. The glutamate decarboxylase (GAD) gene was detected in SH9 by using specific primers. Product of 540 bp was obtained, sequenced, and analyzed (accession number: MW713382). The inferred amino acid sequence was 99.3% identical to *Lactobacillus plantarum* M-6 *gadB* gene. The findings of this study suggest that the marine isolate *E. faecium* SH9 could be used as a novel psychobiotics in the development of GABA rich healthy products.

**Supplementary Information:**

The online version contains supplementary material available at 10.1007/s12602-022-09963-z.

## Introduction

Gamma-aminobutyric acid (GABA) is a non-protein amino acid that can be found in a wide range of microbes, plants, and mammals [1]. Various studies have highlighted that it has a wide range of physiological and psychological activities, as it is predominantly abundant in the brain, where it acts as a major inhibitory neurotransmitter, and it is involved in brain metabolism, memory formation, and cognition. Moreover, GABA helps to regulate physiological diseases such as Alzheimer’s disease, Parkinson’s disease, growth hormones, and protein synthesis in the brain [2]. Dinan et al. [3] used the term “psychobiotics” to describe a new class of probiotics. They are living microorganisms with the potential to improve mental wellness. Psychobiotics improve gastrointestinal (GI) function, and they have antidepressant and anxiolytic capability by affecting central nervous system (CNS) related functions and behaviors via the gut-brain-axis (GBA) through immunological, humoral, neuronal, and metabolic pathways [4]. They may influence neurotransmitters and proteins via production of several neuroactive compounds such as GABA, glutamate, serotonin, and brain-derived neurotrophic factor (BDNF). Both digestive and mental problems are frequently found in the same person. This implies that the central nervous system and the gastrointestinal tract are strongly connected [5]. Therefore, psychobiotics might be considered as delivery vehicles for neuroactive compounds. All of these findings suggest that using psychobiotics might be a feasible alternative for maintaining and restoring mental health, with fewer side effects and a reduced risk of allergies and dependency than psychiatric medications.

Glutamate decarboxylase (GAD) is a cytoplasmic enzyme that is required for the bioconversion of GABA. It can be produced by strains that have the GAD gene [6]. While several lactic acid bacteria (LAB) have the potential to generate GABA, little is known about the genes involved in GABA synthesis in their genomes [7]. The screening of GABA-producing LAB has recently received a lot of attention and research as many LAB are probiotics that offer a variety of health benefits for humans, including the capacity to synthesize and enhance the bioavailability of nutrients, improve digestive tract health, boost the immune system, and fight pathogenic microorganisms [8].

A variety of GABA-producing LAB strains have been isolated from different sources including kimchi [9], dairy products [10], and fermented food [11]. *Lactobacillus*, *Enterococcus*, *Leuconostoc*, *Pediococcus*, *Propionibacterium*, and *Weissella* are among the most common and significant GABA-producing bacteria [6]. Many studies have shown that LAB belonging to the genera *Lactobacillus* and *Lactococcus* produce the highest amount of GABA [2, 6], but just a few studies on *Enterococcus* sp. have been published. LAB are widely dispersed in nature and may be found in a range of environments. In humans, animals, and marine organisms, they are an important part of the gut microbiota [12]. Marine LAB are rich source of novel bioactive compounds as they differ from their terrestrial counterparts in terms of traits and biological activities. Their ability to survive under marine physicochemical conditions boosts their competitiveness compared to their terrestrial counterparts. The key difference between marine and terrestrial LAB is the high endurance of marine LAB to harsh environmental conditions, which may explain the distinctive features of marine LAB by-products [13]. Therefore, the current investigation was carried out to assess the capacity of new marine *Enterococcus* sp. isolate to generate considerable amount of GABA and investigate its potential as a psychobiotic.

## Material and Methods

### Sample Collection and Isolation of LAB

A total of 5 different shrimp samples (*Aristaeomorpha foliacea*, *Marsupenaeus japonicas*, *Squilla mantis*, *Metapenaeus monoceros*, and *Penaeus semisulcatus*) were collected from the Mediterranean Sea, Alexandria, Egypt. The collected samples were stored in sterile bottles, kept in an icebox, and transported to the laboratory. The entire gut of shrimp samples was dissected out. Each gut sample was cultivated in De Man Rogosa and Sharpe (MRS) broth (Neogen, Lansing, MI, USA) then incubated anaerobically at 37 °C for 24 h. The cultivated samples were serially diluted (10^−1^ to 10^−5^) with distilled water, cultured on MRS agar plates, and incubated anaerobically at 37 °C for 24 h. After incubation, the purification of separate colonies was carried out several times by streak-plating on new plates of the same medium. Gram staining was conducted, and Gram-positive isolates were selected.

### Catalase Test

Overnight cultures of isolates were grown on MRS agar anaerobically at 37 °C for 24 h. Two drops of 30% hydrogen peroxide (H_2_O_2_) were added on the bacterial cultures on a glass slide. The catalase test showed positive reaction by the formation of bubbles that indicate the production of catalase enzyme by the test bacterium (catalase positive). Therefore, the isolates, which did not gave gas bubbles (catalase negative), were selected for subsequent activities [14].

### KOH Test

The isolates were grown on MRS agar anaerobically at 37 °C for 24 h. A drop of 3% aqueous KOH was placed on a clean slide. Visible cells from fresh cultures were mixed to the KOH using a sterile loop. The isolates, which did not give a viscid product, were detected as Gram-positive bacteria [15]. Purified Gram positive, catalase negative, non-motile, and non-spore forming strains were selected as presumptive LAB and kept in 20% glycerol solution (v/v) and stored at − 20 °C for further studies.

### Screening for GABA Production

The ability of the obtained 17 presumptive LAB isolates to transform L-glutamic acid to GABA had been demonstrated using thin-layer chromatography (TLC), the isolates were grown in MRS broth containing 1% monosodium glutamate (MSG) at 37 °C for 48 h, and then the culture broth was centrifuged at 2000 × g for 15 min at 4 °C. GABA in the supernatant was qualitatively detected by TLC on silica gel plates (Sigma, Germany). Briefly, 4 µL of the supernatant was spotted on the TLC plate. TLC was conducted using solvent mixture of n-butanol:acetic acid:water (5:2:2, v/v/v), and GABA (Loba, India) was used as a standard. The chromatogram was viewed after spraying with a 2% ninhydrin solution and developing at 105 °C for 5 min, then the retention factor (rf) was calculated according to the equation rf = distance traveled by spot/distance traveled by solvent [16]. The strain which gave a spot at the same rf value as that of the standard GABA indicated that the isolate is a GABA producer and the diameters of developed GABA spots for the GABA-producing strains were measured [17].

### Phenotypic and Biochemical Identification of Isolate SH9

The isolate SH9 was selected as a high GABA producer in the rest of the study. Overnight culture of isolate SH9 was inoculated in the Gram positive (GP) cards and run the cards on the VITEK 2 compact microbial identification system version 07.01 (BioMérieux, France) (Through Mabaret El-Asafra laboratories, Egypt) for biochemical identification and characterization of isolate SH9. It is a completely automated method for bacterial cell identification. It performs a range of biochemical tests on the cells, including sugar fermentation, enzyme hydrolysis, and antibiotic resistance. Moreover, the typical cell shape and size of isolate SH9 were observed using a scanning electron microscope (SEM) **(**JSM-5300—JEOL, Japan), available through the Faculty of Science, Alexandria University.

### Molecular Identification and Phylogenetic Analysis of Isolate SH9

The total genomic DNA of isolate SH9 was extracted and purified according to the manufacturer instructions of the DNA extraction kit (QIAGEN, USA) from overnight culture grown in MRS. The prepared DNA was loaded after mixing with the loading dye in 1% agarose gel prepared in TEA buffer with 1 μg/mL ethidium bromide (Sigma, USA), and the voltage was applied (90 v/cm) for electrophoresis after soaking in TEA buffer. The obtained bands were visualized using the UV transilluminator (Bio-Rad, USA).

The PCR product for SH9 16 s rRNA gene was performed and sequenced by Applied Biotechnology Co., Egypt, for molecular identification of isolate SH9. The obtained sequence was submitted to the National Center for Biotechnology Information (NCBI) GenBank under the accession number MW217575. Moreover, the sequence was analyzed using BLASTn tools available through NCBI which revealed that isolate SH9 has 99.44% similarity with *Enterococcus faecium* strain KUHS13 (accession no. AP022341.1).

Isolate SH9 16 s rRNA gene sequence was further aligned through the ClustalW tool with related species, and the phylogenetic tree was constructed using Maximum Likelihood technique through www.phylogeny.fr.

### Assessment of Probiotic Potential of Isolate SH9

#### Hemolytic Activity

Isolate SH9 was cultured on blood agar plates (supplemented with 7% human blood) followed by incubation at 37 °C for 24 h. Blood agar plates were examined for any signs of hemolysis such as appearance of clear zones of hydrolysis around the colonies (β-hemolysis), partial hemolysis with green zones around colonies (α-hemolysis), or no zones around colonies (γ-hemolysis) [18].

#### Gelatinase Activity

The gelatinase activity of isolate SH9 was examined according to Su et al. [19]. Briefly, isolate SH9 was grown on agar plates containing 3% gelatin (Loba Chemie, India) at 37 °C for 24 h. After incubation, the cultures were flooded with a saturated solution of ammonium sulfate. The presence of a transparent halo around colonies and gelatin precipitates indicated the gelatinase activity.

#### Phenol Tolerance

The tolerance of isolate SH9 to phenol was carried out as described by Aswathy et al. [20]. Briefly, 1% (v/v) of overnight culture of SH9 was inoculated into MRS broth medium supplemented with different phenol concentrations (0.2 and 0.5 g phenol/100 mL) or without phenol and incubated anaerobically at 37 °C for 24 h. Then, the optical density (OD) was measured using a spectrophotometer (Unico, USA) at 620 nm.

#### Antioxidant Activity by DPPH Radical Scavenging Assay

The 1-diphenyl-2-picrylhydrazyl (DPPH) free radical scavenging activity of SH9 was measured quantitatively according to the method described by Lee et al. [16]. Briefly, 500 μL of ethanolic DPPH solution (0.4 mmol) was mixed vigorously with 500 μL of CFS of SH9, or deionized water (as a control) and incubated at 37 °C in the dark for 1 h. The absorbance of the mixture was measured using a spectrophotometer (Unico, USA) at 517 nm. The ascorbic acid was used as a standard, and the scavenging activity was calculated according to the following equation:

Scavenging activity (%) = [1 − (As − Ab) / Ac] × 100.

Where Ab, Ac, and As are the absorbance of the blank (ethanol and sample), the control (DPPH and deionized water), and the sample (DPPH and sample), respectively.

#### Resistance to Low pH

The resistance of isolate SH9 to low pH values was carried out according to Nawaz et al. [21]. The isolate was inoculated (1% v/v) into sterile MRS broth initially adjusted to different pH values (2, 3, 4, and 6.5) with 0.1 N hydrochloric acid and incubated at 37 °C. The absorbance was monitored at hourly intervals for 6 h using a spectrophotometer (Unico, USA) at 620 nm.

#### Bile Salt Tolerance

The effect of different concentrations of bile salts on the growth of isolate SH9 was tested using the method described by Yavuzdurmaz [22]. An overnight culture of SH9 (1% v/v) was inoculated in aliquots of 15 mL sterile MRS broth containing different concentrations of bile salts (0, 0.1, and 0.3% (w/v)) and incubated at 37 °C for 4 h. The absorbance of the culture was monitored using a spectrophotometer (Unico, USA) at 620 nm at hourly intervals.

#### Antimicrobial Activity

The antimicrobial activity of the selected isolate SH9 against different bacterial pathogens including Gram-negative bacteria (*Pseudomonas aeruginosa* ATCC 9027, *Escherichia coli* ATCC 8739, *Klebsiella pneumonia* ATCC 13883, *Vibrio damsela*, *Vibrio fluvialis*, *Pseudomonas fleurescence* DSM 50090, *Aeromonas hydrophila* NRRL 914) and Gram-positive bacteria (*Staphylococcus aureus* ATCC 25923, *Bacillus subtilis* ATCC 6633, *Enterococcus faecalis* ATCC 29212, *Streptococcus agalactiae* CCM 6187) was estimated by the agar well-cut diffusion method [23]. Nutrient agar plates were prepared and inoculated with 1% (v/v) of each pathogenic strain. Wells of 8-mm diameter were cut in solidified agar using a sterile cork borer and filled with 100 µL of filter sterilized CFS of SH9. After incubation of inoculated plates at 37 °C for 24 h, the antimicrobial activity was evaluated by measuring the clear zone diameter around each well.

#### Antibiotic Susceptibility

Susceptibility of SH9 to different antibiotics was determined based on Kirby-Bauer disc diffusion method [24]. Briefly, different antibiotic discs were placed onto the surface of MRS agar plates inoculated with isolate SH9. The plates were incubated at 37 °C or 24 h. After incubation, the development of inhibition zone around each disc was reported indicating the sensitivity of SH9 to the tested antibiotic. The antibiotics used were erythromycin (E 15), tetracycline (TE 30), nalidixic acid (NA 30), ampicillin (AM 10), oxacillin (OX 1), ofloxacin (OFX 5), cephradine (CE 30), ceftriaxone (CRO 30), amoxicillin (AX 25), Piperacillin/Tazobactam (TPZ 110), and Vancomycin (VA 30).

## Anti-inflammatory Effect

### Human Red Blood Cell Stabilization Method

The in vitro anti-inflammatory activity of the selected isolate SH9 was assessed using the human red blood cell (HRBC) membrane stabilization method according to Vane and Botting [25]. An equal volume of sterilized Alsever solution (2% dextrose, 0.8% sodium citrate, 0.5% citric acid, and 0.42% sodium chloride in water) was mixed with human blood. The blood was then centrifuged at 2000 × g for 20 min, and packed cells were separated. The packed cells were washed with isosaline (0.85%, pH 7.2), and a 10% v/v suspension was made with isosaline. This HRBC suspension was used for the estimation of anti-inflammatory property. Diclofenac sodium, 1 mm of sample, and aspirin were separately mixed with 1 mL of phosphate buffer (0.15 M, pH 7.4), 2 mL of hypo saline (0.36%), and 0.5 mL of HRBC suspension. Two-milliliter distilled water was used as the control instead of the sample. The assay mixture was incubated at 37 °C for 30 min and centrifuged at 3000 rpm for 20 min. The supernatant liquid was decanted, and the hemoglobin content in the supernatant solution was measured using a spectrophotometer at 560 nm. The percentage of hemolysis was evaluated by assuming the hemolysis produced in the control as 100%.

The percentage of hemolysis was calculated by using the following formula:

$$\% \;Hemolysis=\frac{OD \;of \;test }{OD \;of \;control} \times 100$$

The percentage of HRBC membrane stabilization or protection was calculated by using the following formula:

$$\% \;Protection = 100 -\frac{OD \;of \;test }{OD \;of \;control} \times 100$$

### Lactose Fermentation

The lactose fermentation capacity of SH9 was detected according to Houssam et al. [26]. Briefly, an overnight culture of SH9 was inoculated in sterilized fermentation medium composed of (gL) peptone 10, NaCl 15, phenol red 0.018, and lactose 5, and the final pH was adjusted to 7.0, then the culture was incubated at 37 °C for 24 and 48 h. Change in color from red to yellow indicates positive lactose fermentation.

### Growth on Methylene Blue

Isolate SH9 was tested for its ability to grow on methylene blue [27]. Overnight culture of the isolate SH9 was inoculated into MRS broth medium supplemented with 0.3% methylene blue and incubated at 37 °C for 24 h. The absorbance of the culture was monitored using a spectrophotometer (Unico, USA) at 820 nm.

### Molecular Characterization of the GAD Gene

The primers from highly conserved regions of GAD, CoreF/CoreR (5′ CCTCGAGAAGCCGATCGCTTAGTTCG 3′ and 5′ TCATATTGACCGGTATAAGTGATGCCC 3′, respectively), were used to amplify genes for GABA-synthesizing enzymes in isolate SH9 total DNA according to Siragusa et al. [28]. Briefly, 50 μL of PCR mixture was prepared containing 200 μM of each dNTP, 1 μM of both primers, 2 μM MgCl_2_ and 2 U of DNA *Taq* polymerase, and 50 ng of DNA. The PCR reaction was performed using the Biometra thermocycler (Germany). The reaction conditions included 5-min denaturation at 94 °C followed by 35 cycles of 94 °C for 30 s, 55 °C for 30 s, then 72 °C for 45 s, and finally 72 °C for 5 min was used for the last cycle. The PCR product was separated in 1.5% agarose gel by electrophoresis as described above.

The PCR product sequencing was carried out through Applied Biotechnology Co., Egypt, and the obtained sequence was submitted to the NCBI GenBank under the accession number MW713382.

### Quantitative Evaluation of GABA

For quantitative measurement of GABA concentration produced by SH9, 10 μL of culture cell free supernatants was subjected to derivatization following the method described by Jo et al. [29]. High performance liquid chromatography (HPLC) analysis was carried out for the derivatized samples using an Agilent 1260 series. The separation was carried out using Eclipse Plus C18 column (4.6 mm × 250 mm i.d., 5 μm). The mobile phase consisted of buffer (sodium phosphate dibasic and sodium borate), pH 8.2 (A), and ACN:MeOH: H2O 45:45:10 (B) at a flow rate 1.5 mL/min at 40 °C. The mobile phase was programmed consecutively for 40 min in a linear gradient of 0–100%. The same process was applied to GAPA standard and to create a calibration curve for eight standard GABA solutions (0.156, 0.313, 0.625, 1.25, 2.5, 5.0, 10.0, and 20.0 gL).

## Statistical Analysis

Data are represented as mean ± standard deviation of three independent experiments. The analysis was carried out using one-way analysis of variance (ANOVA) through Microsoft Excel 2010 software at the significance level of *P* < 0.05.

## Results

### Isolation, Preliminary Characterization of Bacterial Strains

A total of 17 presumptive LAB isolates were isolated and purified on MRS medium; they were characterized by being Gram positive, non-spore former, non-motile, and catalase negative. Among them, 10 isolates were cocci, and 8 were rod shaped.

### Screening for GABA Production

The 17 isolates were tested for the ability to produce GABA from L-glutamic acid by cultivation in MRS medium supplemented with 1% MSG and incubated at 37 °C for 48 h. Then, they were examined by TLC to detect GABA production (Supplementary Fig. S1). Ten isolates (namely, SH1, SH3, SH4, SH6, SH7, SH8, SH9, SH10, SH11, and SH13) gave clear GABA spot at rf of 6.1 corresponding to the standard GABA and were able to convert MSG (rf = 5.4) to GABA after 48 h of incubation at 37 °C. The rest of the isolates did not produce GABA from MSG. The diameters of developed GABA spots for the GABA-producing strains ranged from 5 to 9 mm. Isolate SH9 gave the largest GABA spot (9 mm), so it was selected as a high GABA-producing strain and used for the rest of the study.

### Biochemical Identification

The isolate SH9 was further characterized biochemically by the VITEK 2 system (BioMérieux, France) as described in Table 1. The VITEK 2 characterization of isolate SH9 revealed certain biochemical properties, including lactose fermentation, resistance to four antibiotics, Polymixin, Bacitracin, Novobiocin, and Optochin, as well as the ability to grow in 6.5% NaCl. The isolate SH9 was identified biochemically as *E. faecium* with 95% probability. Moreover, isolate SH9 showed tolerance to phenol up to 0.5% and ability to grow at 0.3% methylene blue.

**Table 1:** Biochemical characterization of SH9 marine bacterial isolate using the VITEK 2 microbial identification system (BioMérieux, France)

**Test**	**Result**	**Test**	**Result**
D-amygdalin (AMY)	−	D-galactose (dGAL)	+
Phosphatidylinositol phospholipase c (PIPLC)	−	D-ribose (dRIB)	+
D-xylose (dXYL)	−	L-lactate alkalinization (ILATK)	−
Arginine dihydrolase 1 (ADH1)	+	Lactose (LAC)	+
Beta-galactosidase (BGAL)	+	N-acetyl-d-glucosamine (NAG)	+
Alpha-glucosidase (AGLU)	−	D-maltose (dMAL)	+
Ala-phe-pro arylamidase (APPA)	−	Bacitracin resistance (BACI)	+
Cyclodextrin (CDEX)	+	Novobiocin resistance (NOVO)	+
L-aspartate arylamidase (AspA)	−	Growth in 6.5% NaCl (NC6.5)	+
Beta galactopyranosidase (BGAR)	−	D-mannitol (dMAN)	−
Alpha-mannosidase (AMAN)	−	D-mannose (dMNE)	+
Phosphatase (PHOS)	−	Methyl-b-d-glucopyranoside (MBdG)	+
Leucine arylamidase (LeuA)	−	Pullulan (PUL)	−
L-proline arylamidase (ProA)	−	D-raffinose (dRAF)	−
Beta glucuronidase (BGURr)	−	O/129 resistance (comp.vibrio.) (O129R)	+
Alpha-galactosidase (AGAL)	−	Salicin (SAL)	+
L-pyrrolydonyl-arylamidase (PyrA)	+	Saccharose/sucrose (SAC)	−
Beta-glucuronidase (BGUR)	−	D-trehalose (dTRE)	+
Alanine arylamidase (AlaA)	−	Arginine dihydrolase 2 (ADH2s)	+
Tyrosine arylamidase (TyrA)	−	Optochin resistance (OPTO)	+
D-sorbitol (dSOR)	−	Urease (URE)	−
Polymixin b resistance (POLYB)	+		

### Molecular Identification of SH9

**Fig. 1:**
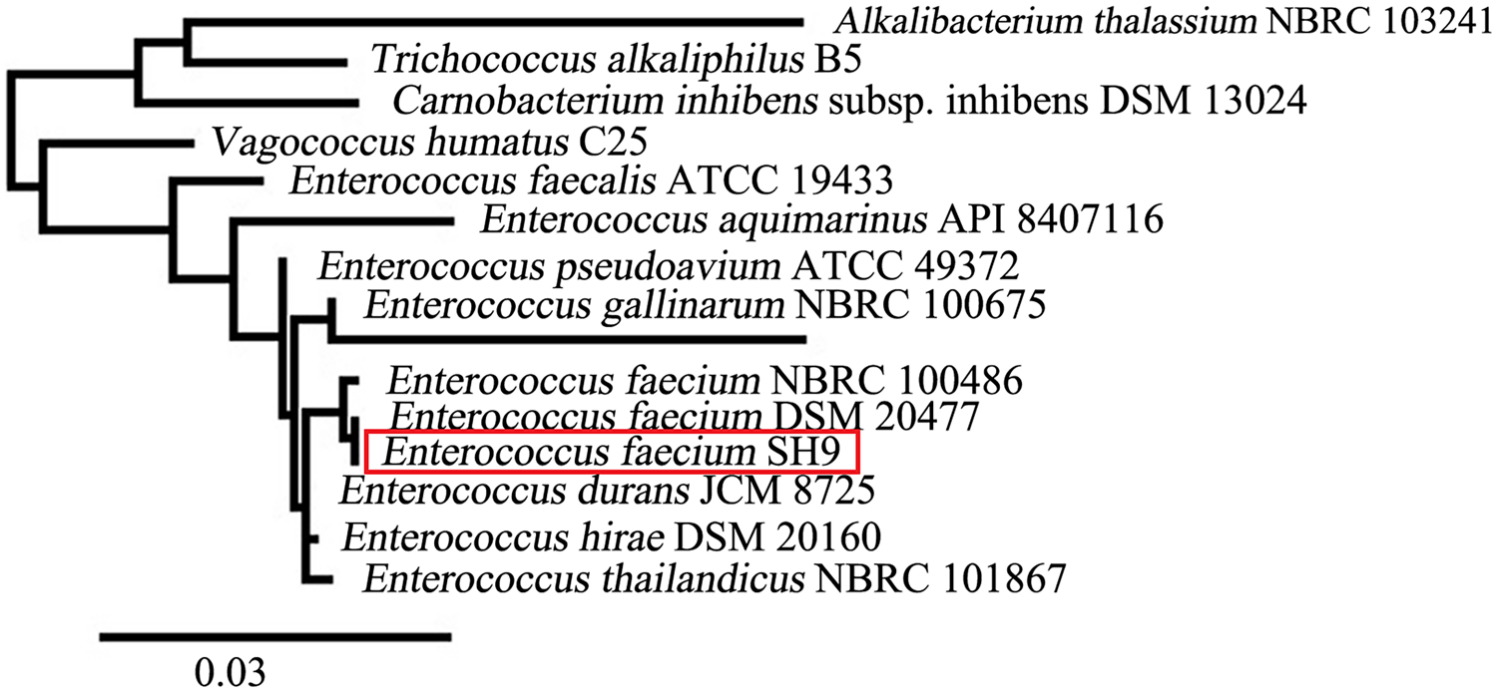
Phylogenetic tree based on the 16S rRNA partial gene sequence of the marine isolate SH9 identified as *E. faecium* by BLAST (NCBI) (submitted to the GenBank under accession number MW217575)

The identification of the marine isolate SH9 was further confirmed by molecular identification through 16S rRNA gene sequencing analysis, and BLAST software of the NCBI was used to carry out sequence homology search. SH9 was identified as *E. faecium* with similarity percentage of 99%, and the sequence was submitted to the GenBank under the accession number MW217575. The phylogenetic relation of the marine isolate SH9 and its close relatives in the NCBI database is represented in Fig. 1.

### Evaluation of Probiotic Potential of Isolate SH9

#### Hemolytic and Gelatinase Activities

Hemolytic activity is one of the significant safety criteria often used to estimate potential probiotic strains. The selected marine isolate SH9 did not show any clear zone or greenish around their colonies on the blood agar plate which implies non-hemolytic activity (Supplementary Fig. S2). At the same time, isolate SH9 showed no gelatinase activity, as it did not show any precipitation of gelatin or halos around cells.

#### Antibiotic Susceptibility

The bacterial isolate SH9 was studied for its antibiotic susceptibility based on the disc diffusion method. The development of inhibition zone around each disc was reported indicating the susceptibility of isolate SH9 to the tested antibiotic. The results in Table 2 indicate that isolate SH9 was resistant to 5 types of antibiotics Erythromycine, Nalidixic Acid, Ampicillin, Ceftriaxone, and Piperacillin/Tazobactam out of 11 clinically important antibiotics that have been tested, while it was sensitive to 6 tested antibiotics.

**Table 2:** Antibiotic susceptibility of selected strain SH9

**Antibiotic**	**Result**
Erythromycine (15 μg)	R
Tetracycline (30 μg)	S
Nalidixic acid (30 μg)	R
Ampicillin (10 μg)	R
Oxacillin (1 μg)	S
Ofloxacin (5 μg)	S
Cephradine (5 μg)	S
Ceftriaxone (30 μg)	R
Amoxicillin (25 μg)	S
Piperacillin/tazobactam (110 μg)	R
Vancomycin (30 μg)	S

*R* Resistant, *S* Sensitive

#### Resistance to pH

**Fig. 2:**
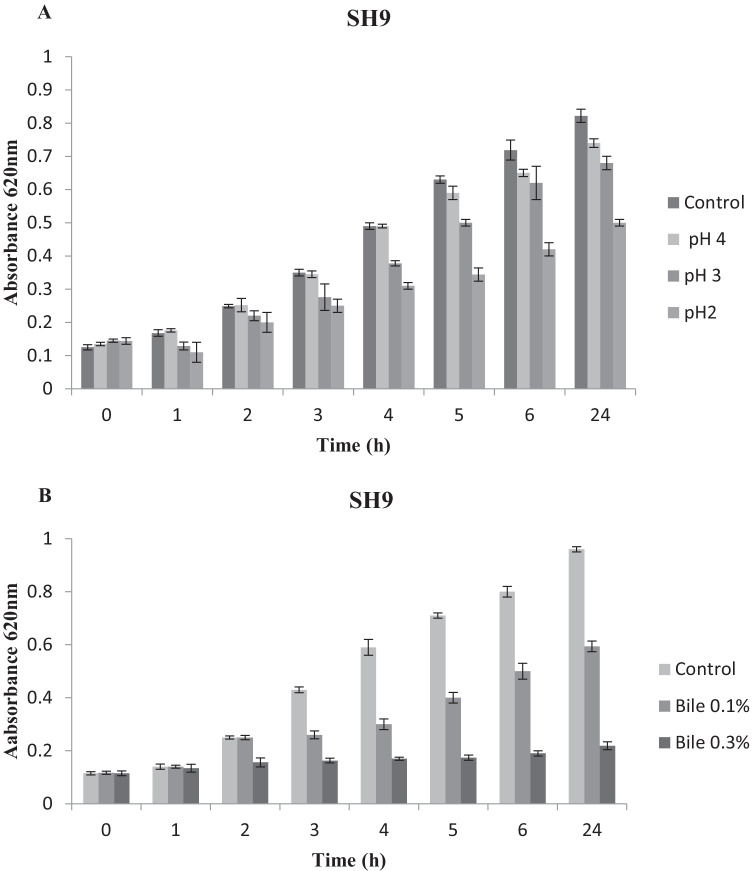
(**A**) Effect of different pH values (2, 3, 4, and control 6.5) and (**B**) different bile salt concentrations (0, 0.1, and 0.3% w/v) on the growth of the marine isolate SH9 at 37 °C for 24 h

The isolate SH9 was grown in different pH values to evaluate its ability to withstand low pH simulating the pH of the stomach which is evaluated to be around 3 and the stay time about 4 h [30]. The results indicated in Fig. 2A showed that the isolate SH9 was able to withstand pH 4 and 3 for 4 h as indicated by the increase in the absorbance with survival rates of 100% and 77%, respectively. At the same time, isolate SH9 was able to survive and continued to grow at slower rate at pH 2 for 4 h with survival rate of 63%, and it continued to grow as observed after 24 h.

#### Bile Salt Tolerance

The ability of isolate SH9 to survive and grow in the presence of various bile salt concentrations was investigated. The average bile salt concentration in the intestinal tract is variable, but it is estimated to be around 0.3% w/v, with a proposed stay period of 4 h [31]. The isolate SH9 was able to withstand the 0.1% bile concentration as indicated by the increase in optical density from 0.1 to 0.5 in 6 h. At the higher bile salt concentration 0.3%, SH9 was able to survive, but the growth was slowed down with slight increase in the optical density which continued to increase as observed after 24 h **(**Fig. 2B).

#### Antimicrobial Activity

The antimicrobial activity of SH9 CFS was determined against 11 pathogenic strains using agar well-cut diffusion method. The results indicated that SH9 cell free supernatant has strong antimicrobial activity against the entire tested pathogens, and the diameter of inhibition zones ranged from 1.16 to 2.43 cm (Table 3). The highest antimicrobial activity was recorded against the Gram-positive bacteria *S. aureus* ATCC 25923 (2.4 cm), and the lowest activity was recorded against the gram negative bacteria *E. coli* ATCC* 8739* (1.16 cm).

**Table 3:** Antimicrobial activity of the marine isolate SH9 CFS against different indicator pathogens

**Indicator pathogen**	**Inhibition zone diameter (cm)**
*Pseudomonas aeruginosa ATCC 9027*	1.6 ± 2.719
*Escherichia coli ATCC 8739*	1.16 ± 0.057
*Klebsiella pneumonia ATCC 13883*	1.33 ± 0.057
*Vibrio damsel*	2.33 ± 0.152
*Vibrio fluvialis*	2.26 ± 0.230
*Pseudomonas fleurescence DSM 50090*	1.73 ± 0.461
*Aeromonas hydrophila NRRL 914*	1.43 ± 0.115
*Staphylococcus aureus ATCC 25923*	2.43 ± 0.057
*Bacillus subtilis ATCC 6633*	2.36 ± 0.152
*Enterococcus faecalis ATCC 29212*	1.96 ± 0.305
*Streptococcus agalactiae CCM 6187*	1.93 ± 0.057

#### Antioxidant Activity

The CFS of isolate SH9 was investigated for its antioxidant activity using DPPH assay test. The CFS showed a very promising scavenging activity of 90.36% as indicated by the decrease in the concentration of the DPPH radical compared to the scavenging activity of the control ascorbic acid recorded to be 74.42%. Moreover, the CFS showed strong anti-inflammatory activity as indicated by protection% of 91.04.

### Molecular Characterization of the GAD Gene

The GABA content of *E. faecium* SH9 CFS was evaluated by HPLC to be 0.97 gL. Therefore, it was screened for the presence of GAD genes. Primers designed from highly conserved region of the GAD genes gave a PCR product of approximately 540 bp (Supplementary Fig. S3). The obtained fragment was sequenced, and the predicted amino acid sequence is “LATFCQTYMEPEAVELMKDTLAKNAIDKSEYPRTAEIENRCVNIIANLWHAPDDEHFTGTSTIGSSEACMLGGLAMKFAWRKRAQAAGLDLNAHRPNLVISAGYQVCWEKFCVYWDVDMHVVPMDEQHMALDVNHVLNYVDEYTIGIVGIMGIT.”

**Fig. 3:**
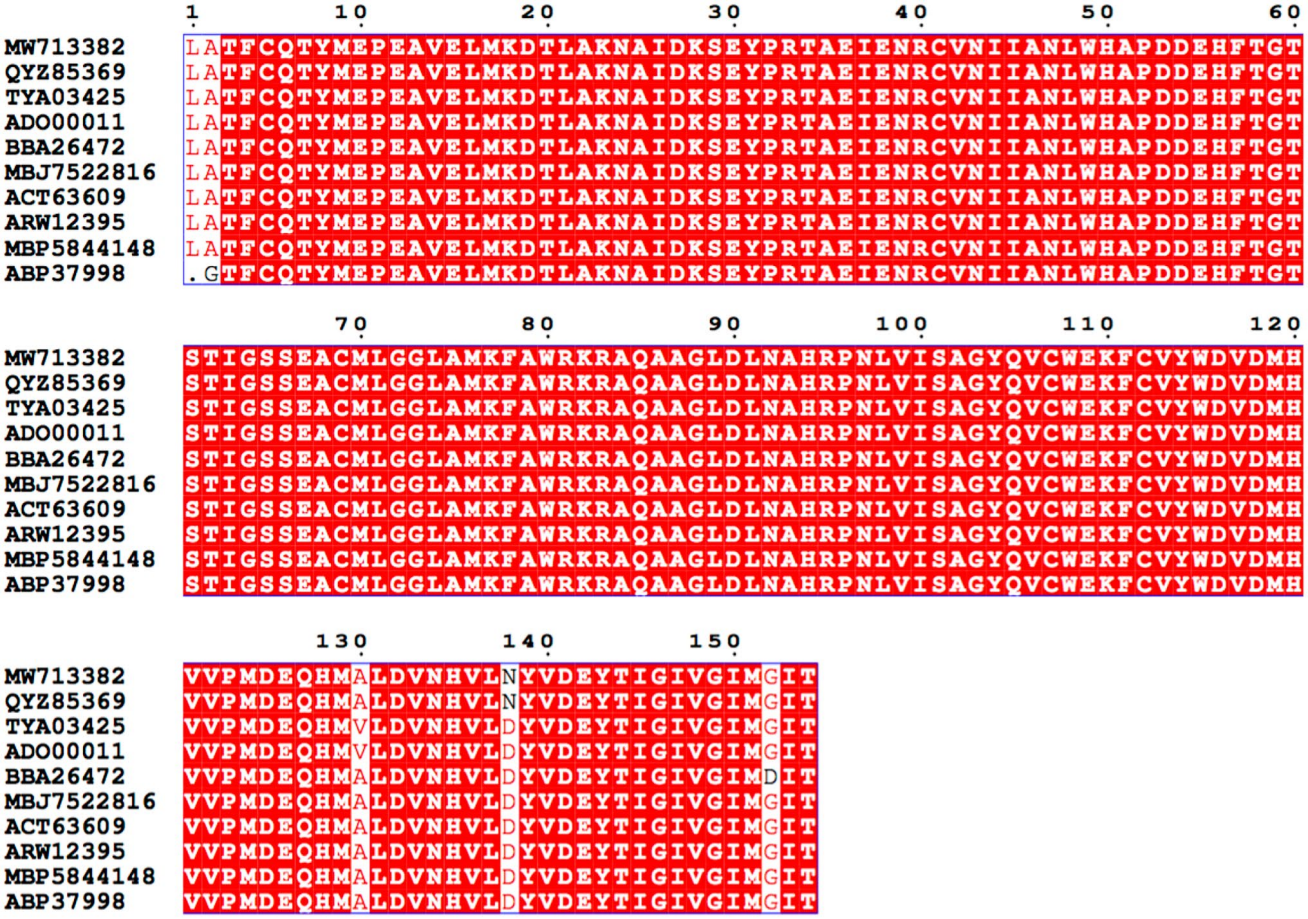
Multiple sequence alignment between ten glutamate decarboxylase protein sequences from the database and isolate *E. faecium* strain SH9 glutamate decarboxylase (MW713382). The red shade refers to identical amino acids or sequences in the defined positions. The alignment was made by ClustalW and visualized using ESPript 3.0 available through https://espript.ibcp.fr/ESPript/cgi-bin/ESPript.cgi

It was analyzed using NCBI blastx software and showed 99.3% similarity to *gadB* of *Lactobacillus* *plantarum* M-6 (Accession number KU214639.1) in the database. Relativity distance of the internal GAD gene fragment of *E. faecium* SH9 with the eight most relevant sequences of the GAD gene from the database is shown in Fig. 3. The sequence of *E. faecium* SH9 GAD gene was deposited in GenBank under the accession number MW713382.

## Discussion

The core for a rational scientific selection of next-generation psychobiotics strains that may treat various psychiatric disorders in the future is the investigation of novel strains with high neuroactive potential metabolites that have specific mechanisms able to mediate specific physiological responses and have a positive effect on the host’s mental state [3].

Microbial-derived GABA has attracted the attention of many researchers in the latest years, particularly from LAB strains isolated from various sources such as human intestinal tracts, fermented food, and beverages [32]. Marine microorganisms are a relatively new and understudied source of novel bioactive compounds [33, 34]. Therefore, in this study, 17 marine presumptive LAB were isolated and screened for GABA productio; 64.7% of the obtained isolates were able to produce GABA as indicated by TLC analysis. The marine isolate *E. faecium* SH9 was selected as a high GABA-producing strain, and it was evaluated for the main recommended criteria for safety consideration as a probiotic [35].

The food industry has to screen different probiotics capable of manufacturing GABA since this form of GABA production delivers natural GABA, a bioactive ingredient that alters health aspects and provides consumers with novel and appealing food products [32].

*E. faecium* SH9 showed no signs of hemolysis. The non-hemolytic activity of *Enterococcus* sp. was previously reported as De Vuyst et al. [35] indicated that *Enterococcus* species (*E. faecium*, *E. faecalis*, *E. casseliflavus*, *E. durans*, *E. gallinarum*, and *E. hirae*) isolated from different origins exhibited non-hemolytic activity. In addition, Pieniz et al. [36] reported the same result with *E. durans* strain LAB18s isolated from Minas Frescal cheese (typical Brazilian soft cheese). At the same time, SH9 was able to withstand 0.2% phenol concentration. The ability of bacterial strain to survive the primarily phenols and toxic metabolites produced during digestion process is also an essential probiotic feature. Bacteria in the gut can deaminate some aromatic amino acids derived from dietary or endogenously synthesized proteins, resulting in the formation of phenols which can exert bacteriostatic characteristics [37].

SH9 showed resistance to 5 tested antibiotics and resistance to 5 types. This could be advantageous to be able to develop antibiotic/probiotic combination therapies [38]. If the probiotic is sensitive to antibiotics in medicines and food, it will be eliminated easily from the intestinal tract and will no longer be functional. As a result, probiotics survival in the presence of some antibiotics is essential [39].

SH9 exhibited Vancomycin (VA)30 susceptibility, which is an important feature because Vancomycin is frequently used as a last resort antibiotic and is one of the most efficient antibiotics against multidrug-resistant bacteria that cause clinical infections.

Because of safety issues, *Enterococcus* strains should be used with caution in the food business [40]. Aspri et al. [41] investigated the technological and safety aspects of *Enterococcus* strains isolated from raw donkey milk, finding that the majority of isolates were vancomycin-resistant and a few had virulence genes.

Moreover according to Khanlari et al. [40] in many LAB species, the antibiotic resistance gene is placed on the chromosome, which is innate and non-transferable making antibiotic resistance genes from probiotic bacteria unlikely to be transferred to other pathogenic strains in the gut [42].

The marine isolate *E. faecium* SH9 showed promising ability to withstand low pH values of (4, 3, and 2) with survival rate of 100, 77, and 63% respectively. These results are in accordance with Pimentel et al. [43] who isolated a total of 73 strains of enterococci from Terrincho cheese and identified them as *E. faecium* (45.2% of isolates), *E. durans* (39.7%), and *E. faecalis* (2.74%) and indicated that most strains of *E. faecium* and *E. durans* were tolerant to acidic conditions. At the same time, Sabna et al. [39] reported that *E. faecium* BS5 showed high survival rate at pH (4, 3, and 2) of 78%, 78%, and 25% respectively. While, at pH 1 *E. faecium* BS5 was not able to grow. At the same time, the isolate *E. faecium* SH9 was able to survive and tolerate 0.1% and 0.3% bile salt concentrations, in agreement with Sabna et al. [39] who reported that *E. faecium* BS5 was able to tolerate different bile concentrations. The bile salts are so harmful to living cells as they alter the membrane structure of the cell. The probiotic strain tolerance to high bile salt concentrations is attributed to a specific enzyme called bile salt hydrolase (BSH); this enzyme helps to hydrolyze the conjugated bile salts and reduce its toxicity [44], or certain dietary components can reduce bile’s toxic effect on microorganisms. A prior exposure to low pH for 3 h could potentially result in the induction of bile resistance [21].

During its evolution as a gut commensal, *E. faecium* must have developed mechanisms to perceive, respond to, and tolerate bile as a successful colonizer of the intestinal tract. In a mouse peritonitis model, two genetic loci (gls33-glsB and gls20-glsB1) that encode Gls-like proteins in *E. faecalis* and *E. faecium* were previously identified as being important in bile resistance and pathogenicity. *E. faecium* also has bile salt hydrolase activity, which is provided by the BSH gene-encoded protein (accession no. AY260046) [45].

Low pH tolerance may be less significant than the capacity to survive in the small intestine and resist its environment. Some studies have shown that acid sensitive strains can be buffered through the stomach, but in order to have a positive impact on the host, the probiotic strain must be able to withstand degradation by hydrolytic enzymes and bile salts present in the intestine, as well as survive and colonize in the small intestine, which makes this property critical in the selection of new probiotic strains [36].

SH9 showed promising antimicrobial activity against all tested pathogenic strains. This is very important in probiotic strain selection as it must have the capability to eliminate the competitors. LAB produces a number of antimicrobial compounds that give them a significant advantage over other pathogenic bacteria in the gut [46].Variations in strain and growing circumstances, on the other hand, can impact the production of the antimicrobial compounds [47]. These antimicrobial agents include organic acids such as lactic and acetic acid, fatty acids, acetoin, hydrogen peroxide, diacetyl, and most importantly, inhibitory peptides known as bacteriocins. The bacteriocins produced by enterococci are referred to as enterocins. Numerous enterocins have been identified and characterized from enterococci [48].

The ability of LAB to synthesize GABA is a strain-dependent trait that varies widely between strains and is controlled by the *gadB* and *gadC* genes [49]. *L. plantarum*, *Lactobacillus* *paracasei* [28], and *Lactococcus* *lactis* are the most common GABA-producing strains [49]. However, the potential of *Enterococcus* sp. to produce GABA is significantly less explored, and previously, the GABA-producing *Enterococcus* strains were rarely reported, such as *Enterococcus* *avium* and *E. faecium* [50].

GABA is produced as a result of the irreversible decarboxylation of L-glutamate by the GAD enzyme, which is pyridoxal 5-phosphate dependent enzyme that catalyzes the α-decarboxylation [51]. In the presence of L-glutamate, GAD is an intracellular enzyme that helps LAB tolerate acidic stress by reducing the proton concentration in the cytoplasm. In the intracellular GAD system, glutamate is imported into the cells via the GABA antiporter, decarboxylated by intracellular GAD enzyme to synthesize GABA, and finally GABA is exported from the cells via the antiporter [52].

The highest reported amount of GABA production was produced by the strain *Lactobacillus* *buchneri* WP2001, and it reached up to 70 g/L [53]. Lim et al. [50] isolated *E*. *faecium* as a GABA producer from Kimchi, a Korean fermented food, and they reported high level of GABA production of 1.56 mM.

In this study, the marine isolate *E. faecium* SH9 successfully produced up to 0.97 g/L of GABA. In accordance with this result, Khanlari et al. [40] reported that the fermentation process of milk by *E. faecium* MDM21 successfully increased the amount of GABA from 18 to about 50 mg/L. Shan et al. [54] reported production of 314.5 mg of GABA per 100 mL of yogurt, and Seo et al. [55] reported production of 231 mg/100 mL of GABA in fermented milk. These variations in GABA concentration might be influenced by many factors such as medium composition, temperature, pH, and inoculum size [56, 57].

GAD genes are discovered in the gut microbiome, and GABA-producing probiotic strains affect health and behavior in animal models. GABA’s sleep-inducing impact has also been demonstrated in animal studies, which revealed that a group fed GABA-containing milk produced more melatonin and serotonin, both of which are regarded as sleep inducers [58]. The ability to produce GABA may be a significant trait in the selection of bacterial strains as psychobiotics, as it is thought to be involved in the interaction of gut microbiota with macroorganisms. In addition, NaCl is one of the most significant additions for food preservation; therefore, the capacity to withstand salty conditions is a desired characteristic which makes marine psychobiotics more suitable for food and industrial applications.

## Conclusion

The current study reveals that a GABA-producing bacterium, *E. faecium* SH9, was successfully isolated from marine samples and found to have high GABA-producing abilities, with a GABA concentration of 0.97 g/L assessed by HPLC in SH9 CFS. SH9 has probiotic potential as well, showing no signs of hemolysis and the ability to survive at low pH and high bile salt concentrations. It also has antimicrobial properties against highly pathogenic microorganisms and can thrive in a 6.5% NaCl. SH9 CFS also demonstrated promising anti-inflammatory and antioxidant properties. The GAD gene responsible for GABA production was detected in SH9 using specific primers. A 540-bp product was sequenced and analyzed (accession number: MW713382). The amino acid sequence deduced was 99.3% identical to that of the *L. plantarum* M-6 gadB gene. The findings of this investigation imply that, *E. faecium* SH9, a marine isolate, could be used as a novel psychobiotic to manufacture or expand the industrial value of functional pharmaceutical and food GABA-rich products.

## Supplementary Information

Below is the link to the electronic supplementary material.

Supplementary file1 (DOCX 1402 KB)

## Data Availability

Data available upon request.
