# Supplementary material for: Psychobiotic Potential of Gamma-Aminobutyric Acid–Producing Marine Enterococcus faecium SH9 from Marine Shrimp
Source: Probiotics Antimicrob Proteins. 2022 Jun 25;14(5):934–46. doi: 10.1007/s12602-022-09963-z (PMC9474364; doi:10.1007/s12602-022-09963-z)
Supplement: Supplementary file 1 — Supplementary file1 (DOCX 1402 KB) [file 12602_2022_9963_MOESM1_ESM.docx]

**
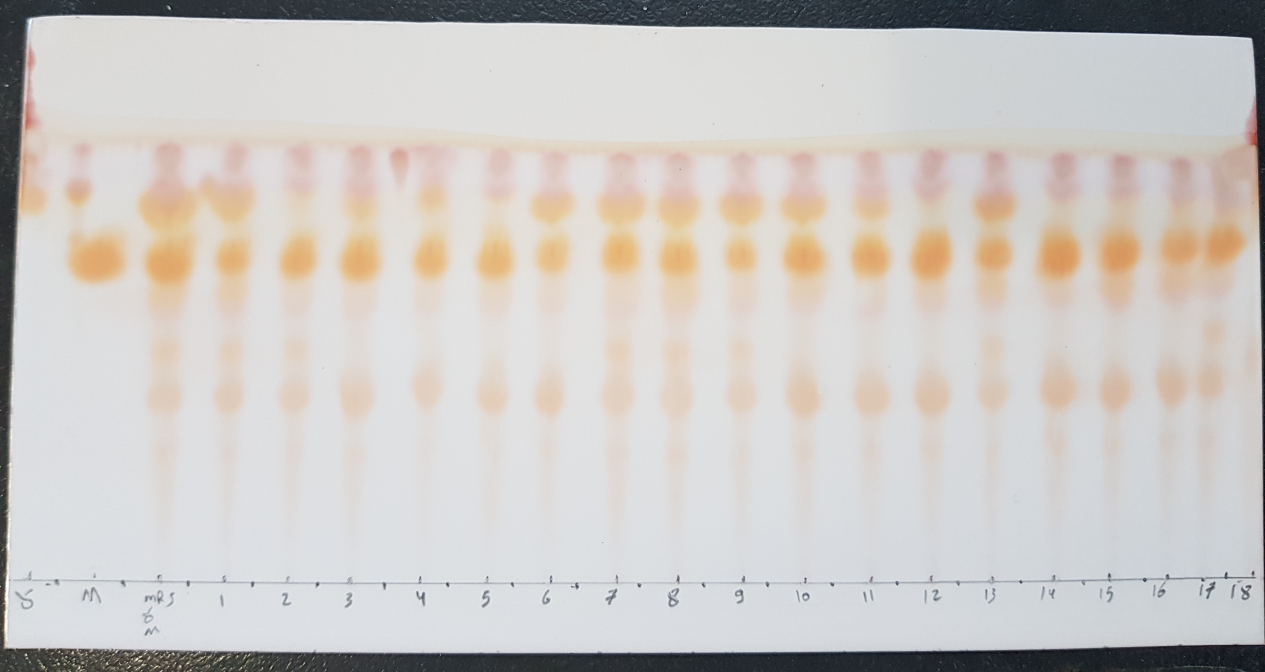
**

**Fig. S1** TLC chromatogram that indicates GABA production by the experimental LAB isolates. Lane 1: MSG (control); lane 2: GABA and MSG standard in MRS followed by the 17 presumptive LAB isolates.


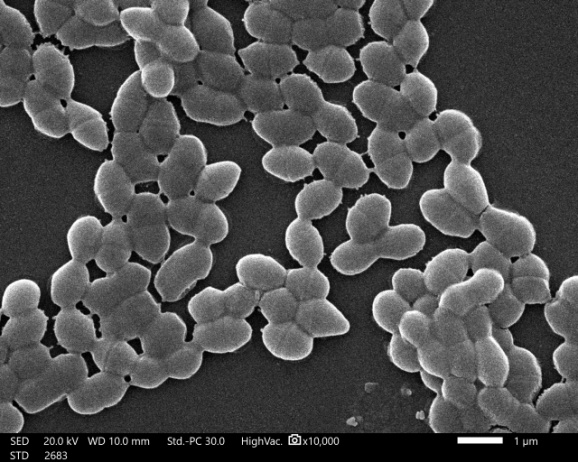

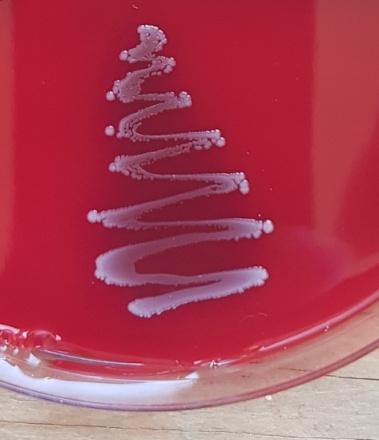


**B**

**A**

**Fig. S2** **(A)** Scanning Electron Microscope (SEM) imaging of SH9 bacterial cells. demonstrates the SH9 isolate characteristic spherical cell shape, size and grouping in clusters using SEM. **(B)** Blood hemolysis test for isolate SH9 after 24 h of incubation at 37 ºC.

**
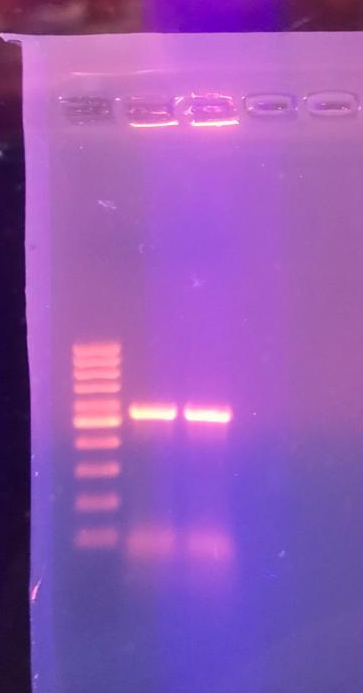
**

**540 bp**

**Fig. S3** Molecular detection of GAD gene in the marine isolates *E. faecium* SH9 using gene specific primers. Lanes 1 represents DNA Ladder, Lane 2 showing clear bands at expected size of GAD gene at 540 bp for isolates *E. faecium* SH9 and Lane 3 is a positive control (A GABA producing strain).
